# Correction: Nosi et al. MET Exon 14 Skipping: A Case Study for the Detection of Genetic Variants in Cancer Driver Genes by Deep Learning. *Int. J. Mol. Sci.* 2021, *22*, 4217

**DOI:** 10.3390/ijms27020986

**Published:** 2026-01-19

**Authors:** Vladimir Nosi, Alessandrì Luca, Melissa Milan, Maddalena Arigoni, Silvia Benvenuti, Davide Cacchiarelli, Marcella Cesana, Sara Riccardo, Lucio Di Filippo, Francesca Cordero, Marco Beccuti, Paolo M. Comoglio, Raffaele A. Calogero

**Affiliations:** 1 Department of Molecular Biotechnology and Health Sciences, University of Torino, 10126 Torino, Italy; vladimir.nosi@unito.it (V.N.); alessandri.luca1991@gmail.com (A.L.); maddalena.arigoni@unito.it (M.A.); 2 Candiolo Cancer Institute-FPO, IRCCS, 10060 Candiolo, Italy; melissa.milan@ircc.it (M.M.); silvia.benvenuti@ircc.it (S.B.); 3 Telethon Institute of Genetics and Medicine (TIGEM), 80078 Pozzuoli, Italy; d.cacchiarelli@tigem.it (D.C.); sara.riccardo@ngdx.eu (S.R.); lucio.difilippo@ngdx.eu (L.D.F.); 4 Department of Computer Sciences, University of Torino, 10149 Torino, Italy; francesca.cordero@unito.it (F.C.); marco.beccuti@unito.it (M.B.); 5 IFOM-FIRC Institute of Molecular Oncology, 20139 Milano, Italy; pcomoglio@gmail.com

The journal’s Editorial Office and Editorial Board are jointly issuing a resolution and removal of the Journal Notice linked to this article [1], as well as an update to the original publication. Following concerns raised about the integrity of one of the peer reviews, the Editorial Office has conducted a post-publication peer review of this article. This process included the recruitment of a new independent reviewer and was supervised by the original Academic Editor to ensure full compliance with MDPI’s Editorial Process (https://www.mdpi.com/editorial_process).

As a result of this process, the Academic Editor and the authors have agreed to update the following aspect of this publication:

- Reviewer report 1 was removed from the peer-review record and the new report was uploaded instead (https://www.mdpi.com/1422-0067/22/8/4217/review_report).

Based on the new review report, the following corrections have been made to the original publication:

The “disruption” was replaced with “alteration” in the Introduction section. This modification appears on page 1, paragraph 1. The cluster characterized by the intron 2 coverage peak has been renamed “LINE1–MET–like cluster”, while the remaining clusters are now described as showing canonical MET transcription patterns. The updated naming appears in the legend in Figure 6. References [21,22] have been removed from the manuscript, resulting in a change in the biography. The entire sentence “as well as other generalist CNNs [21,22]” on page 3 was removed.

With this update, the Academic Editor is satisfied that the Editorial Process relating to this article has been completed as per MDPI’s Editorial Process policy. The Editorial Office would like to thank the authors for their collaboration during this process.

With this correction, the order of some references has been adjusted accordingly. The authors state that the scientific conclusions are unaffected. This correction was approved by the Academic Editor. The original publication has also been updated.
