# Supplementary material for: MET Exon 14 Skipping: A Case Study for the Detection of Genetic Variants in Cancer Driver Genes by Deep Learning
Source: Int J Mol Sci. 2021 Apr 19;22(8):4217. doi: 10.3390/ijms22084217 (PMC8072630; doi:10.3390/ijms22084217)
Supplement: Supplementary file 1 [file ijms-22-04217-s001.zip › ijms-1178062-supplementary.pdf]

Supplementary data

MET exon 14 skipping: a case study for the detection of genetic variants in cancer driver genes by deep learning.

Nosi V*[1], Alessandrì L*[1], Milan M[2], Benvenuti S[2], Cordero F[3], Beccuti M[3], Comoglio P[2§], Calogero RA[1#§]

Supplementary Figure 1S: METΔ14 false positive detected in corpus uteri. a) WT MET from A549 RNAseq sample (33 million reads), 27152 reads mapping on MET locus, b) METΔ14 from NCI-H596 RNAseq sample (27 million reads), 24850 reads mapping on MET locus, c-f) False METΔ14 in corpus uteri samples, g) False METΔ14 in heart/mediastinum/pleura samples, h-l) False METΔ14 in kidney samples, m) False METΔ14 in skin samples.

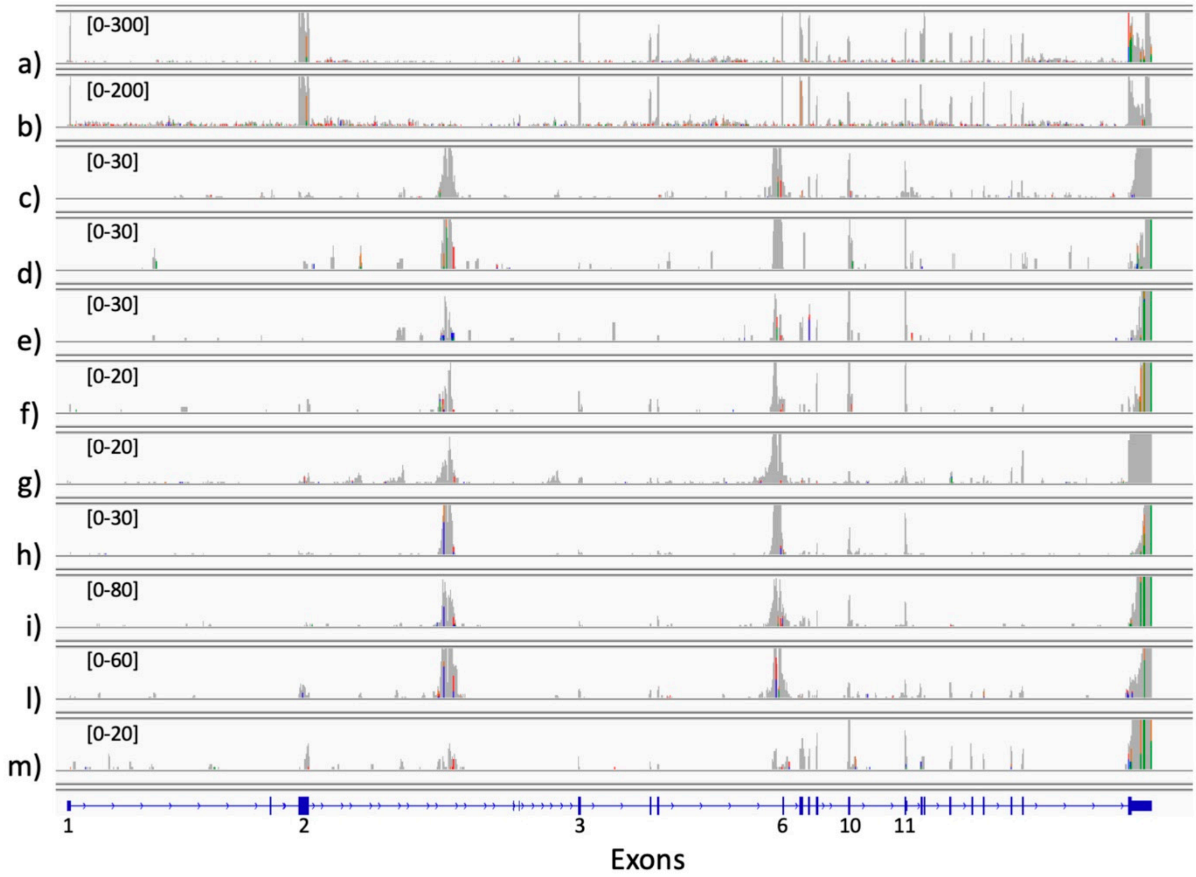

Supplementary Table 1S: Set of COSMIC census genes present in the ExonSkipDB. The first column is the set of genes associated to articles describing the presence of a skipping event in that gene linked to cancer.

Symbol	PMID	Symbol								
ALK	28665006	A1CF	ASXL1	BRD3	CDK6	CTNND1	EML4	FBXO11	GPC3	KIF5B
APC	33670833	ABI1	ASXL2	BRD4	CEP89	CTNND2	EPAS1	FCGR2B	GPHN	KIT
BAP1	33681728	ABL1	ATF1	BUB1B	CHCHD7	CUL3	EPHA3	FCRL4	HERPUD1	KMT2A
BRCA1	32761968,32203205	ABL2	ATIC	CACNA1D	CHD2	CUX1	EPS15	FES	HIF1A	KMT2C
BRCA2	29707112	ACSL3	ATM	CALR	CHD4	DCTN1	ERBB3	FGFR1	HIP1	KMT2D
BRIP1	30230034	ACSL6	ATP1A1	CAMTA1	CHIC2	DDB2	ERBB4	FGFR1OP	HMGA1	KNSTRN
BTK	12854903	ACVR1	ATP2B3	CANT1	CIC	DDR2	ERCC2	FGFR2	HMGA2	KTN1
CDH1	26674321,33465060	ACVR2A	ATR	CARD11	CLIP1	DDX10	ERG	FGFR4	HNRNPA2B1	LARP4B
CHEK2	31349801	AFF1	ATRX	CASP8	CLTC	DDX5	ESR1	FH	HOOK3	LASP1
ERBB2	33214226	AFF3	AXIN1	CASP9	CLTCL1	DDX6	ETNK1	FIP1L1	HSP90AB1	LATS2
ETV6	30940639	AFF4	AXIN2	CBFA2T3	CNTRL	DEK	ETV1	FLI1	IDH2	LCK
EXT1	23629877	AKT1	BAX	CBFB	COL1A1	DGCR8	ETV4	FLNA	IGF2BP2	LEF1
EZH2	27090213	AKT2	BAZ1A	CBLB	COL3A1	DNM2	ETV5	FLT3	IKBKB	LIFR
GLI1	11032829	ALDH2	BCL11B	CBLC	CPEB3	DNMT3A	EWSR1	FLT4	IKZF1	LMNA
JAK2	32826247,25617626	ANK1	BCL2L12	CCNE1	CREB1	DROSHA	EXT2	FNBP1	IL6ST	LMO1
MDM4	26595814	AR	BCL3	CD209	CREBBP	EBF1	EZR	FOXP1	IRF4	LRIG3
MET	33313283,30600919,30459450,33495574,33497758,33618059,33249657,31564914,30459450,33660106,33641723,33638808,33667719,33643443,33533182,33545388,33540494	ARAF	BCL6	CD274	CRNKL1	ECT2L	FAM131B	FSTL3	ITGAV	LRP1B
MLH1	28381181,19767099	ARHGAP26	BCL9L	CD28	CRTC1	EED	FANCA	FUBP1	KAT7	LZTR1
MUTYH	20628285	ARHGAP5	BCLAF1	CD74	CRTC3	EGFR	FANCC	FUS	KDM5A	MALT1
NF2	10401006	ARHGEF10L	BCOR	CD79B	CSF1R	EIF1AX	FANCD2	GAS7	KDM5C	MAP2K1
NOTCH2	24574459	ARHGEF12	BCORL1	CDC73	CSF3R	EIF3E	FANCG	GMPS	KDM6A	MAP2K2
PIK3R1	28561224	ARID1A	BCR	CDH11	CSMD3	EIF4A2	FAS	GNAQ	KDR	MAP2K4
PTCH1	30997576,27561271	ARID1B	BIRC3	CDH17	CTCF	ELF3	FAT1	GNAS	KDSR	MAP3K1
SUFU	27840902	ARID2	BIRC6	CDK12	CTNNA2	ELK4	FAT4	GOLGA5	KEAP1	MAP3K13
TP53	16647569	ARNT	BMP5	CDK4	CTNNB1	ELN	FBLN2	GOPC	KIAA1549	MAPK1
		MAX	NCOR2	PAX8	PPP6C	REL	SH2B3	STIL	TRRAP	
		MED12	NDRG1	PBRM1	PRCC	RET	SH3GL1	STRN	TSC1	
		MEN1	NF1	PCM1	PRDM1	RFWD3	SIRPA	SUZ12	TSC2	
		MITF	NFATC2	PDCD1LG2	PRDM16	RNF43	SLC34A2	SYK	U2AF1	
		MLF1	NFE2L2	PDE4DIP	PRDM2	ROBO2	SLC45A3	TBL1XR1	UBR5	
		MLLT1	NFIB	PDGFRA	PREX2	RPL10	SMAD2	TBX3	USP6	
		MLLT10	NFKB2	PDGFRB	PRKACA	RPL22	SMAD3	TCEA1	USP8	
		MLLT3	NFKBIE	PER1	PRKCB	RPL5	SMARCA4	TCF3	VAV1	
		MSH2	NIN	PHF6	PRPF40B	RPN1	SMARCD1	TCF7L2	VTI1A	
		MSI2	NOTCH1	PICALM	PTK6	RSPO2	SMARCE1	TEC	WAS	
		MSN	NPM1	PIK3CA	PTPN13	RUNX1	SMC1A	TERT	WIF1	
		MTOR	NRG1	PIK3CB	PTPN6	SBDS	SMO	TFEB	WNK2	
		MUC16	NSD1	PIM1	PTPRB	SDHA	SND1	TFG	WRN	
		MYB	NT5C2	PLCG1	PTPRT	SDHC	SNX29	TFRC	XPC	
		MYH11	NTHL1	PML	QKI	Sep-05	SPECC1	THRAP3	XPO1	
		MYH9	NTRK1	PMS1	RABEP1	Sep-06	SPEN	TMPRSS2	ZBTB16	
		MYO5A	NTRK3	PMS2	RAC1	Sep-09	SRC	TNC	ZEB1	
		NAB2	NUMA1	POLD1	RAD17	SET	SRGAP3	TNFAIP3	ZFHX3	
		NACA	NUP214	POLE	RAD21	SETBP1	SRSF3	TP63	ZMYM2	
		NBEA	NUP98	POLG	RAF1	SETD1B	SS18	TPM4	ZMYM3	
		NBN	PABPC1	POLQ	RALGDS	SETD2	STAG1	TPR	ZNF384	
		NCKIPSD	PAFAH1B2	POT1	RANBP2	SETDB1	STAG2	TRAF7	ZNF521	
		NCOA1	PALB2	PPFIBP1	RAP1GDS1	SF3B1	STAT3	TRIM24	ZNRF3	
		NCOA2	PATZ1	PPM1D	RB1	SFRP4	STAT5B	TRIM33	ZRSR2	
		NCOR1	PAX5	PPP2R1A	RBM10	SGK1	STAT6	TRIP11		

Supplementary Table 2S: samples in TCGA bronchus and lung predicted as METΔ14 using different training configurations. METΔ14 score ≤ 0.1 predicts a skipped event. NN quality score ranges between 1 and 0, where 1 indicates an optimal NN performances.

NN training	sample	METΔ14 score	NN quality score	MET reads	sample mapped reads	sample unmapped reads	skipping manual check
k-mer counts whole MET	4f7de6bd-55ea-43c3-a04a-1c57d647d4d3	0.001162738	0.9978775	34087	44562703.5	855013.5	TRUE
k-mer counts whole MET	9b056fb3-407d-4adc-9720-25b9d3055be7	0.004646361	0.99833864	11391	78987772	1998091.5	FALSE
k-mer counts whole MET	ce9c0d52-542b-4586-99b0-4c409582e76d	0.008714974	0.99846476	39918	124595862.5	2162904.5	FALSE
k-mer counts whole MET	0f8f62da-daf0-4a59-9740-b03bd0e0ea3b	0.008776099	0.9991946	5951	69446926.5	1678773	FALSE
k-mer frequency exons 13-15 MET	c9032291-b671-4280-96d2-7a5806e66886	2.04E-05	0.6346185	59071	90868832	1714512	TRUE
k-mer frequency exons 13-15 MET	56b38e12-3495-4419-8df6-d3504b190357	0	0.7081907	44070	136788143	43362420	TRUE
k-mer frequency exons 13-15 MET	911fdc9d-3de5-493d-98c7-f567072ca1d5	1.84E-05	0.438411	34418	78565359	2271496	TRUE
k-mer frequency exons 13-15 MET	4f7de6bd-55ea-43c3-a04a-1c57d647d4d3	0.004308641	0.6003467	34087	44562703.5	855013.5	TRUE

k-mer frequency exons 13-15 MET	92650f66-f8b3-49c8-a1fc-04fba0ffb6fe	0	0.33768573	32307	60202242	1021761	TRUE
k-mer frequency exons 13-15 MET	15750b18-0522-4186-8809-e7e3e7e4f6f7	0	0.42051643	28130	87516179.5	3472464.5	TRUE
k-mer frequency exons 13-15 MET	9d5fde39-1fc9-4079-b095-bdc94bbb3679	2.98E-08	0.61794007	26170	60892059.5	1328162.5	TRUE
k-mer frequency exons 13-15 MET	8cac0361-6973-4ae6-a34a-58f355085ec0	0.26194692	0.6702909	19455	59264871	2258010	TRUE
k-mer frequency exons 13-15 MET	eaf344c1-a5e8-4893-b17e-4a0dbb1bf2de	1.17E-05	0.66456187	19083	41267020.5	752423	TRUE
k-mer frequency exons 13-15 MET	7f841dfd-84db-4a14-8709-9831dba468dd	0.007866561	0.4141181	213987	82285020.5	8009125	FALSE
k-mer frequency exons 13-15 MET	4500c5e4-665c-40f8-8cf2-c98b48bf91b3	0	0.3985763	150600	55852402	3757212	FALSE
k-mer frequency exons 13-15 MET	faf59a82-243a-49ea-bd8c-597fc39aaccf	0	0.4208542	149532	51748284	821212	FALSE
k-mer frequency exons 13-15 MET	a1905555-3d81-4446-b90b-e5d1d2519fc0	0.506146	0.47563305	132515	93308372.5	1975561.5	FALSE
k-mer frequency exons 13-15 MET	c0c54d0d-21c5-4342-a718-7168e1f7c6f2	0	0.4733004	128205	78811903.5	1409834	FALSE
k-mer frequency exons 13-15 MET	3c4e102d-5c5b-4b90-84a0-67b8ee465103	0.10779613	0.5216947	92166	100329080.5	2104674	FALSE
k-mer frequency exons 13-15 MET	49b48e8f-6f90-4ebf-bacb-1c8f1c19fc27	0	0.42244694	66055	111587657	2849114	FALSE
k-mer frequency exons 13-15 MET	9ac13263-c20f-4530-bd4f-d20118d20db0	5.96E-08	0.4589822	66004	48535694	820903.5	FALSE
k-mer frequency exons 13-15 MET	3aa4bd0a-d550-45b0-943c-c8d60d30fef4	0	0.43359128	65061	91858435.5	1193531.5	FALSE
k-mer frequency exons 13-15 MET	afb174de-1a55-4291-86cf-31149eea106d	0.1952982	0.46828112	57896	77608179.5	1675932	FALSE
k-mer frequency exons 13-15 MET	1895e19e-becd-42df-89c8-d5b1dff12900	0.4330656	0.45584062	52073	104546884	1463551	FALSE
k-mer frequency exons 13-15 MET	1e0021fe-8d1e-4705-b668-d59082a19a0e	0	0.41332984	51520	139597004.5	2247079	FALSE
k-mer frequency exons 13-15 MET	d29989bb-7681-4820-b969-aada70be39db	0.026408255	0.7107893	49439	139471432.5	2267034	FALSE
k-mer frequency exons 13-15 MET	81df7e45-a5f4-436b-a416-c1f5bd0b7d7b	0.002586871	0.4817645	45915	40807001.5	798823	FALSE
k-mer frequency exons 13-15 MET	c5da92a7-03e7-406a-ae2e-716c0a123f49	0.002166063	0.4245238	44497	64494538	1341246	FALSE
k-mer frequency exons 13-15 MET	2aa3ed71-57ec-44ec-a46f-2f91c414f065	3.07E-06	0.42654774	44295	133830180.5	10092829	FALSE
k-mer frequency exons 13-15 MET	77fb6dab-f570-4407-a8ba-3fb7bf650733	0	0.44985628	42151	57488639	876582.5	FALSE
k-mer frequency exons 13-15 MET	16dfa0e8-e7cf-4589-bd99-82944d703695	0	0.41464037	41055	153319272	14320268.5	FALSE
k-mer frequency exons 13-15 MET	2ece65d6-95f0-4767-a61e-afea1d5c081f	0	0.35185537	40783	90245553	1205305	FALSE
k-mer frequency exons 13-15 MET	f76af440-35f6-44a7-9cb7-6ef05f2ccefc	1.04E-06	0.42843267	40302	139215769	2334288.5	FALSE
k-mer frequency exons 13-15 MET	3ae44dc0-6028-4507-9254-f4f25afe23e9	0	0.44031847	38643	36307762.5	728040.5	FALSE
k-mer frequency exons 13-15 MET	ae2a91da-cf5e-44f6-98a7-68048b5cbe70	0	0.41499674	38308	71380872.5	1741235.5	FALSE
k-mer frequency exons 13-15 MET	88a8c964-9016-4d53-b7bc-19f643dbb9d5	5.07E-07	0.3430768	37085	120917506	6939020	FALSE
k-mer frequency exons 13-15 MET	c49020ee-ccb5-4c6d-9f08-c557ff6c9fe4	0	0.46568942	36830	100844566	1470598	FALSE
k-mer frequency exons 13-15 MET	54235af3-7abe-46ae-9304-f6b77283b612	0	0.3953657	34383	102992987.5	8705807	FALSE
k-mer frequency exons 13-15 MET	d8a06aad-0658-4ecc-abcc-c5c598a3757e	0	0.413293	34141	113664922.5	1602384.5	FALSE
k-mer frequency exons 13-15 MET	52c684a9-86af-452b-9270-2326e76297d4	0	0.46620095	31782	73043283	4216208.5	FALSE
k-mer frequency exons 13-15 MET	95960889-f586-4e92-88e1-3f38773eba65	0	0.39559016	31762	106277260.5	3042996.5	FALSE
k-mer frequency exons 13-15 MET	9a04f107-a775-46a3-853d-0d1f004464d8	0	0.37244126	31392	72216140.5	1405896	FALSE
k-mer frequency exons 13-15 MET	fed45eec-0500-427c-8c46-df96610786e7	0.000868261	0.42000765	30536	58375861	1098778.5	FALSE
k-mer frequency exons 13-15 MET	72e9fed2-7e36-4ca3-a0c3-5f021bf94e67	0.021142334	0.45462963	29997	80357761.5	2330063	FALSE
k-mer frequency exons 13-15 MET	87fc72eb-6c02-4bcc-8b45-fb6de8fd15cf	0	0.3471099	28588	56184822	839292.5	FALSE
k-mer frequency exons 13-15 MET	a5435b7d-7365-46b9-a238-d1a1a8c1fd18	3.55E-05	0.43290186	28030	113577323	2224418.5	FALSE
k-mer frequency exons 13-15 MET	f45c0334-854d-487b-994c-d17688ff2341	0	0.37980986	27682	98862033	1155534	FALSE
k-mer frequency exons 13-15 MET	fc2065f9-7e23-4a28-b084-b810d1a87009	4.77E-07	0.47523004	26875	84775792	2589971.5	FALSE
k-mer frequency exons 13-15 MET	cb3524ce-ac32-4ac0-8244-2a36237a3b27	0.002302229	0.46727285	25658	93800839	4006990	FALSE
k-mer frequency exons 13-15 MET	b9703fc4-4834-4f11-b316-278fe19a0b7f	0	0.42219284	24979	99261176	2312882	FALSE
k-mer frequency exons 13-15 MET	b170515e-38b9-4c78-b9f7-8d79a99e7ca8	0	0.44390014	24948	113872841.5	2027134.5	FALSE

k-mer frequency exons 13-15 MET	e64c7950-36a9-43eb-999a-53d3e8e06f32	0	0.44836974	24832	114655664	2772213	FALSE
k-mer frequency exons 13-15 MET	e1b6127d-6908-4b72-ab62-c57883f56800	0	0.41094878	24294	103168657.5	6228618	FALSE
k-mer frequency exons 13-15 MET	d89dd4a8-3573-434a-aa2e-000b662b2d77	0.000345886	0.44228142	24093	103782912.5	4560812	FALSE
k-mer frequency exons 13-15 MET	8118b882-54d5-45fa-985b-dd0cf075d111	0	0.43454012	24038	89102483	2803429.5	FALSE
k-mer frequency exons 13-15 MET	3df91e6e-5a33-4f85-8ad3-b19257b847a8	0	0.37833697	23862	101491797	1451727.5	FALSE
k-mer frequency exons 13-15 MET	8c023b1f-d64f-410c-89ad-95537889ff2d	0	0.41056025	23694	91770420	2425348.5	FALSE
k-mer frequency exons 13-15 MET	9e08eb7f-a831-437d-9146-714a0303b833	7.75E-07	0.4661902	23432	64254999	1659247.5	FALSE
k-mer frequency exons 13-15 MET	b0ec29ac-ab53-4e5a-ae93-dd9ba28cbc4d	0	0.7366078	23408	92255167	3485235	FALSE
k-mer frequency exons 13-15 MET	dfff0629-41f9-449e-8ec5-0dae9c9018a1	1.03E-05	0.39416534	23135	131738863.5	2978214.5	FALSE
k-mer frequency exons 13-15 MET	8b525cb8-15c0-4404-847e-7dcfcd9e1cb3	0	0.39097697	22826	80110803	2846774.5	FALSE
k-mer frequency exons 13-15 MET	f0a7639f-4215-42f9-9625-8c488769029a	0.45235044	0.45030108	22307	97587933.5	3011049	FALSE
k-mer frequency exons 13-15 MET	5a11d7d0-8a47-4b18-8892-2188d56b8642	1.19E-06	0.7497631	21313	40848856	617674	FALSE
k-mer frequency exons 13-15 MET	d037e7a7-4df8-4db5-99be-dc0f0b6d0d3a	0	0.41706824	20970	126359609.5	6007250.5	FALSE
k-mer frequency exons 13-15 MET	81e4f97e-6756-4173-9336-fc7801bd03c7	0	0.41236764	20814	128164018.5	3168741.5	FALSE
k-mer frequency exons 13-15 MET	73e7a49e-87fd-4809-a076-1394a1a619a2	2.21E-06	0.3548656	20408	141553456.5	5672381	FALSE
k-mer frequency exons 13-15 MET	0a420947-598b-47de-a254-93019042daef	0	0.452678	20095	37813444	2850196.5	FALSE
k-mer frequency exons 13-15 MET	f08a284a-681e-4a7b-bee0-508fbaec55ff	0	0.45339435	20088	90466629.5	2107851	FALSE
k-mer frequency exons 13-15 MET	c8ccea38-7660-45bf-8344-87a8e5b1287b	0.006163925	0.4692268	20042	65443224	1534072.5	FALSE
k-mer frequency exons 13-15 MET	48faa87a-9523-4ce7-8e57-43080b3044f1	0	0.44731477	19304	60650862	815096.5	FALSE
k-mer frequency exons 13-15 MET	4f4ce53d-4b77-4266-93ba-3040e2b13468	0.000507951	0.4331196	19035	58742467	1158130	FALSE
k-mer frequency exons 13-15 MET	d6cbd9fc-6e44-46cc-a263-e53212e6229e	0.008203536	0.40848738	18886	119049814	2079067.5	FALSE
k-mer frequency exons 13-15 MET	91b7daf7-dfeb-49df-a3f7-bdbf29a50543	0	0.43229455	18746	106811791	1416549	FALSE
k-mer frequency exons 13-15 MET	7e83b372-ba64-4212-84fd-0e307b030459	2.98E-08	0.4358817	18694	87982849	2188749.5	FALSE
k-mer frequency exons 13-15 MET	49205969-292e-49d1-a9a7-fcc8454488c8	0.005163103	0.43851304	18622	69797336.5	1120730.5	FALSE
k-mer frequency exons 13-15 MET	25a1f5c7-843b-4162-a4ee-11e283f60537	1.49E-07	0.44369146	18404	61594906	1678691	FALSE
k-mer frequency exons 13-15 MET	ac05b23c-cd73-4f6a-81a8-79fdb182627a	0	0.43756267	18359	82592394	1411256	FALSE
k-mer frequency exons 13-15 MET	cbe79a08-97c9-4e4e-8a61-37d229765d31	0.007531226	0.38793322	18245	91690707.5	3621048.5	FALSE
k-mer frequency exons 13-15 MET	ee076693-ee73-47cd-9f86-fcac5c3816db	5.96E-08	0.4635978	18117	53645113.5	1221159.5	FALSE
k-mer frequency exons 13-15 MET	29aa71aa-3138-4f9c-8c25-b39cc26a416d	0	0.42977247	18107	108902013.5	2391606.5	FALSE
k-mer frequency exons 13-15 MET	7c661c1f-c92d-4917-8590-9b78428c8533	0.07932618	0.7292144	18090	46701333.5	1034884.5	FALSE
k-mer frequency exons 13-15 MET	aa2fc42a-8f32-433c-ae8a-40e4840d9bfc	0	0.43607333	18007	56023381.5	1643120	FALSE
k-mer frequency exons 13-15 MET	02b1cdfe-7435-41a2-942d-f29e97d2bc7f	0	0.4211828	17609	116650531.5	2174530.5	FALSE
k-mer frequency exons 13-15 MET	5477f6db-0aa5-431b-9eb8-f0b7b1bd4788	0.044130176	0.46012035	16577	92938638	1329186	FALSE
k-mer frequency exons 13-15 MET	f6115dfc-e6f5-44e8-be44-7723ac199bbb	8.76E-06	0.44035006	16399	73036108	1194292.5	FALSE
k-mer frequency exons 13-15 MET	108d891c-7874-45f5-b90f-1b38c22cfe5c	0	0.4509345	16099	97893193.5	1691953	FALSE
k-mer frequency exons 13-15 MET	7296a01f-877c-47ac-91d7-9a44723de8ed	0.59611183	0.7819192	15823	118275931	3436981	FALSE
k-mer frequency exons 13-15 MET	058b3345-ecce-4056-95cc-450ba173dac9	0.06480482	0.43272218	15692	73887228	1909359.5	FALSE
k-mer frequency exons 13-15 MET	45bfe815-8049-4b5b-9a3d-4d6a72616d4d	2.68E-07	0.45592317	15574	111969468.5	2803178	FALSE
k-mer frequency exons 13-15 MET	ee0793f3-5015-4cac-8db2-7124f2fdff42	0.16254914	0.7441665	14732	73674679.5	3423953	FALSE
k-mer frequency exons 13-15 MET	e20bf59c-256c-4222-9040-386bc0e57a51	0	0.41168103	14617	84710355	2222307	FALSE
k-mer frequency exons 13-15 MET	35bb9c34-aace-48fd-9340-8458372d7be3	0	0.44106996	14556	89309517	4405411	FALSE
k-mer frequency exons 13-15 MET	ad223f64-bd39-4050-a823-f7d663106a5b	0	0.44475886	14424	86397083	1127419.5	FALSE
k-mer frequency exons 13-15 MET	f6fa0e02-a6c3-4632-935d-31074dc1b50b	1.08E-05	0.43211523	14369	86314073.5	1327849	FALSE

k-mer frequency exons 13-15 MET	d566e8f1-7164-4440-974f-d8a0b29d9bc6	0	0.43402886	14072	123077732.5	2512388.5	FALSE
k-mer frequency exons 13-15 MET	56b91343-bd50-4ed9-a6a5-d2e069416e08	0.000516415	0.41572964	13719	70942734	1510199	FALSE
k-mer frequency exons 13-15 MET	3b29b654-2513-49f0-98d3-d7c266d8414e	0	0.40619373	13643	97275991.5	2627013	FALSE
k-mer frequency exons 13-15 MET	27354ff8-6ac7-41c0-842b-f2470bd31d1d	0.000889152	0.4113403	13420	107433989	2286760.5	FALSE
k-mer frequency exons 13-15 MET	ac3494b7-32aa-455f-a9b0-d7a91589e556	0	0.37820816	12973	40578914	1182951.5	FALSE
k-mer frequency exons 13-15 MET	4cfb07bb-c8a1-4588-8f75-2b05566aa458	0	0.40203926	12665	73482011	2315607	FALSE
k-mer frequency exons 13-15 MET	dfc8eb3c-312e-4535-980c-f5fd14c15b57	0	0.33102697	12146	80129698.5	902952.5	FALSE
k-mer frequency exons 13-15 MET	0d92d69a-541c-4775-b247-52d44ed8b187	0	0.4931284	12031	59218622	1221031.5	FALSE
k-mer frequency exons 13-15 MET	54531d92-cc37-47b9-acb4-588d0d66a27a	0	0.39219725	11813	84808563.5	2005338.5	FALSE
k-mer frequency exons 13-15 MET	d0eb4e19-8c96-494e-8d79-fe9b05afe076	0	0.40390953	11790	71605640.5	1538049	FALSE
k-mer frequency exons 13-15 MET	ce4afab3-4f6e-4b88-ad56-5557df5e2139	0	0.39683476	11778	73729276	2234315	FALSE
k-mer frequency exons 13-15 MET	6283be9d-4077-4f2a-a523-f010196f5e4e	0	0.4150509	11726	53941556	1398673.5	FALSE
k-mer frequency exons 13-15 MET	79becab4-a3b2-4035-8dc0-e8326a7b4a39	0	0.3816074	11507	47801573	884443.5	FALSE
k-mer frequency exons 13-15 MET	89c0aa10-be63-419e-9991-5db262c0fd81	3.87E-07	0.46010524	11417	40570310.5	849402.5	FALSE
k-mer frequency exons 13-15 MET	1f560ccd-e207-4e79-a3f4-ec9a956a8791	0	0.40607762	11382	55767096	2236742.5	FALSE
k-mer frequency exons 13-15 MET	a3a72a59-d1b5-4d06-a8c5-65f4010fd0d4	0	0.4061726	11238	66958456	1541630	FALSE
k-mer frequency exons 13-15 MET	70a5549e-b93d-4440-8968-85a0826b3a29	0	0.38679016	11041	95942178.5	2348248.5	FALSE
k-mer frequency exons 13-15 MET	1990e1af-3fe6-4727-a324-9a86f04336bd	1.88E-06	0.3449738	11009	76111677.5	1715189.5	FALSE
k-mer frequency exons 13-15 MET	e5f5aa88-0b0e-4620-8ff4-3316ab3e9cd8	0	0.44548082	9960	85058681.5	1889305.5	FALSE
k-mer frequency exons 13-15 MET	9e9e6d41-ed67-4769-8afd-aa286b9c2d22	0	0.4452252	9794	58618155.5	850993	FALSE
k-mer frequency exons 13-15 MET	850dfca7-2aef-4f86-861a-020e59225848	8.94E-08	0.42548668	9582	35132484	865686	FALSE
k-mer frequency exons 13-15 MET	76a2fc4b-ec9d-434d-96bf-d142bb1cb881	0	0.38740957	9370	48906695.5	592712.5	FALSE
k-mer frequency exons 13-15 MET	97fc45d2-c025-4111-9925-0c1635a32171	0.006449163	0.4493788	9208	71927911	1918489.5	FALSE
k-mer frequency exons 13-15 MET	f94a732c-b382-4584-8564-462500687c09	0	0.42518178	9163	77316732	1883638	FALSE
k-mer frequency exons 13-15 MET	301806f7-e592-4cc1-be8c-664a035a4551	1.24E-05	0.3532794	9116	44779810.5	662344.5	FALSE
k-mer frequency exons 13-15 MET	55646242-65cb-48b8-ae08-6ceb08fb33b7	4.35E-06	0.47437426	9014	61553245	1358515	FALSE
k-mer frequency exons 13-15 MET	4fa39f9c-6863-4186-959d-b060a7d9c581	0.47495693	0.46021995	9000	54675450.5	1270546.5	FALSE
k-mer frequency exons 13-15 MET	ed63448c-0fa2-4377-9802-f10665d50766	0	0.4200727	8731	81942073	1946929.5	FALSE
k-mer frequency exons 13-15 MET	910d472c-1939-4bd3-b293-ff02483c4b79	0	0.44806004	8013	45215374	732391	FALSE
k-mer frequency exons 13-15 MET	5be08aec-828b-4038-a699-ea8b8645bc77	0	0.44790164	7773	77679589.5	4069888.5	FALSE
k-mer frequency exons 13-15 MET	73416b2f-f0ca-4af3-8abc-03010c9bed12	6.65E-05	0.38727385	7686	65520384	1193620	FALSE
k-mer frequency exons 13-15 MET	43ca912a-27ba-4b64-bf12-6a6beb464a86	0.001965314	0.4572315	7642	103534411.5	1613099.5	FALSE
k-mer frequency exons 13-15 MET	66b757f5-36d4-48d2-a37b-a522423126e7	0	0.49366984	7607	80591454	1949902	FALSE
k-mer frequency exons 13-15 MET	fb3d40f2-16d8-408f-bd89-85969e0b80a1	0	0.40378413	7500	43359871.5	982661	FALSE
k-mer frequency exons 13-15 MET	3229ccfa-39b3-49b7-98a0-2d8ba1f07d8e	8.34E-07	0.46533343	7439	94217722	1794210	FALSE
k-mer frequency exons 13-15 MET	e11813c9-fb99-4320-aa8e-6d009e6abd2d	3.01E-06	0.4774029	7423	80079489	1736638	FALSE
k-mer frequency exons 13-15 MET	7003b664-2b95-47f8-b0db-d7c8cdd3c85c	0.14872584	0.45120782	7415	140635884	4364378.5	FALSE
k-mer frequency exons 13-15 MET	cbdbeb39-fd66-4ba8-a36d-8b00c68aee24	0.003278196	0.38209686	7250	54321171	711455	FALSE
k-mer frequency exons 13-15 MET	a63ec3d0-6a36-42f0-a3b5-28d44893c043	0	0.42122582	7079	40665461.5	826640.5	FALSE
k-mer frequency exons 13-15 MET	d7877ffb-2a43-468a-a890-9a4807a548fa	0	0.422462	6845	43592304.5	992310.5	FALSE
k-mer frequency exons 13-15 MET	9792d25b-3830-4808-a47b-071eea851c71	9.27E-06	0.40607238	6505	52720404.5	945556.5	FALSE
k-mer frequency exons 13-15 MET	e8c8e0d5-3b7a-4936-a06d-78fa1c9ebaf9	0	0.40873408	6440	39329781	761141.5	FALSE
k-mer frequency exons 13-15 MET	bbb9c38e-6134-4107-8793-c5b734a8e207	0	0.49958205	6055	75668979.5	1310826.5	FALSE

k-mer frequency exons 13-15 MET	8d6896e4-4534-4026-84e2-decab7b0c561	0.000122696	0.5003371	6036	75726355.5	1830639.5	FALSE
k-mer frequency exons 13-15 MET	c977ad0a-d1ea-4c15-82b0-a1d8f9c24c02	0	0.32081473	5518	82875965	1156254.5	FALSE
k-mer frequency exons 13-15 MET	e6d1567b-49d5-428b-a3d7-cc82481ae1d8	0	0.37478673	5302	74718854.5	2951197.5	FALSE
k-mer frequency exons 13-15 MET	31ef5b4c-de4c-4f3a-a2eb-a46ca5642bee	0.00018838	0.3426908	5130	74906054	1824216	FALSE
Coverage frequency exons 13-15 MET	010a6f81-5601-4fd6-ad42-4d5670d48ff1	0	0.8507528	139031	86536796	1746345	TRUE
Coverage frequency exons 13-15 MET	509b1e8b-8e1e-4d27-b9c7-a13942ae5dc9	0	0.8169447	127554	68401183	1726606	TRUE
Coverage frequency exons 13-15 MET	654c93db-8b88-4619-ae04-e4c561133bc3	0	0.811287	114696	60171126	990399.5	TRUE
Coverage frequency exons 13-15 MET	be2ba5b6-5652-4daa-a29f-e4db98262008	0	0.8196091	58379	65644230.5	1644546	TRUE
Coverage frequency exons 13-15 MET	56b38e12-3495-4419-8df6-d3504b190357	0	0.82740474	44070	136788143	43362420	TRUE
Coverage frequency exons 13-15 MET	911fdc9d-3de5-493d-98c7-f567072ca1d5	0	0.80841327	34418	78565359	2271496	TRUE
Coverage frequency exons 13-15 MET	4f7de6bd-55ea-43c3-a04a-1c57d647d4d3	0	0.7431444	34087	44562703.5	855013.5	TRUE
Coverage frequency exons 13-15 MET	92650f66-f8b3-49c8-a1fc-04fba0ffb6fe	0	0.8132071	32307	60202242	1021761	TRUE
Coverage frequency exons 13-15 MET	5f7c9fc6-f894-4dce-98b0-a9d3a36392e8	0	0.8444487	28641	106888363.5	3169442.5	TRUE
Coverage frequency exons 13-15 MET	15750b18-0522-4186-8809-e7e3e7e4f6f7	0	0.81890565	28130	87516179.5	3472464.5	TRUE
Coverage frequency exons 13-15 MET	9d5fde39-1fc9-4079-b095-bdc94bbb3679	0	0.8005151	26170	60892059.5	1328162.5	TRUE
Coverage frequency exons 13-15 MET	8cac0361-6973-4ae6-a34a-58f355085ec0	0	0.8241724	19455	59264871	2258010	TRUE
Coverage frequency exons 13-15 MET	eaf344c1-a5e8-4893-b17e-4a0dbb1bf2de	0	0.81365246	19083	41267020.5	752423	TRUE
Coverage frequency exons 13-15 MET	c9032291-b671-4280-96d2-7a5806e66886	5.96E-08	0.8233008	59071	90868832	1714512	TRUE
Coverage frequency exons 13-15 MET	2a7a996d-89ab-4717-831f-788461559037	3.51E-05	0.83253515	7560	43568960.5	563888	TRUE
Coverage frequency exons 13-15 MET	eeea8a1f-1967-4a88-8f3e-f746070d62b8	0.012374461	0.85565615	30093	126032419.5	3760419	TRUE
Coverage frequency exons 13-15 MET	e210c2a9-40c7-4f6e-9e5a-d6840c2e3a5c	0.05090207	0.85936856	8684	35173297.5	507258.5	TRUE
Coverage frequency exons 13-15 MET	9b056fb3-407d-4adc-9720-25b9d3055be7	0.02354762	0.819528	11391	78987772	1998091.5	FALSE

Supplementary Table 3S: MET exons

>ENSG00000105976|1|MET
ATGAAGGCCCCCGCTGTGCTTGCACCTGGCATCCTCGTGCTCCTGTTTACCTTGGTGCAGAGGAGCAATGGGGAGTGTAAAGAGGCACTAGCAAAGTCCG
AGATGAATGTGAATATGAAGTATCAGCTTCCCAACTTCACCGCGGAAACACCCATCCAGAATGTCATTCTACATGAGCATCACATTTTCCTTGGTGCCACTA
ACTACATTTATGTTTTAAATGAGGAAGACCTTCAGAAGGTTGCTGAGTACAAGACTGGGCCTGTGCTGGAACACCCAGATTGTTTCCCATGTCAGGACTGC
AGCAGCAAAGCCAATTTATCAGGAGGTGTTTGGAAAGATAACATCAACATGGCTCTAGTTGTCGACACCTACTATGATGATCAACTCATTAGCTGTGGCAG
CGTCAACAGAGGGACCTGCCAGCGACATGTCTTTCCCCACAATCATACTGCTGACATACAGTCGGAGGTTCACTGCATATTCTCCCCACAGATAGAAGAGC
CCAGCCAGTGTCCTGACTGTGTGGTGAGCGCCCTGGGAGCCAAAGTCCTTTCATCTGTAAAGGACCGGTTCATCAACTTCTTTGTAGGCAATACCATAAAT
TCTTCTTATTTCCCAGATCATCCATTGCATTCGATATCAGTGAGAAGGCTAAAGGAAACGAAAGATGGTTTTATGTTTTTGACGGACCAGTCCTACATTGAT
GTTTTACCTGAGTTCAGAGATTCTTACCCCATTAAGTATGTCCATGCCTTTGAAAGCAACAATTTTATTTACTTCTTGACGGTCCAAAGGGAAACTCTAGATG
CTCAGACTTTTCACACAAGAATAATCAGGTTCTGTTCCATAAACTCTGGATTGCATTCCTACATGGAAATGCCTCTGGAGTGTATTCTCACAGAAAAGAGAA
AAAAGAGATCCACAAAGAAGGAAGTGTTTAATATACTTCAGGCTGCGTATGTCAGCAAGCCTGGGGCCCAGCTTGCTAGACAAATAGGAGCCAGCCTGA
ATGATGACATTCTTTTCGGGGTGTTCGCACAAAGCAAGCCAGATTCTGCCGAACCAATGGATCGATCTGCCATGTGTGCATTCCCTATCAAATATGTCAAC
GACTTCTTCAACAAGATCGTCAACAAAAACAATGTGAGATGTCTCCAGCATTTTTACGGACCCAATCATGAGCACTGCTTTAATAGG>ENSG0000105976
|1|MET
GTAGGACCCTCCGAGCCAGGTGAGGGATATAATCTCGTGGTGCGCCGTTTTTTAAGCCGGTCCGAAAAGCGCAATATTCGGGTGGGAGTGACCCGATTTT
CCAG>ENSG00000105976|1|MET
GCAGTTAAAGGTGAAGTGTTAAAAGTTGGAAATAAGAGCTGTGAGAATATACACTTACATTCTGAAGCCGTTTTATGCACGGTCCCCAATGACCTGCTGA
AATTGAACAGCGAGCTAAATATAGAG>ENSG00000105976|1|MET
GCAGGTGACCCGGAGGCCCTCGCCGCCCGCGGCGCCCCGAGCGCTTTGTGAGCAGATGCGGAGCCGAGTGGAGGGCGCGAGCCAGATGCGGGGCGAC
AGCTGACTTGCTGAGAGGAGGCGGGGAGGCGCGGAGCGCGCGTGTGGTCCTTGCGCCGCTGACTTCTCCACTGGTTCCTGGGCACCGAAAG>ENSG000
00105976|1|MET
AGACACGTGCTGGGGCGGGCAGGCGAGCGCCTCAGTCTGGTCGCCTGGCGGTGCCTCCGGCCCCAACGCGCCCGGGCCGCCGCGGGCCGCGCGCGCCG
ATGCCCGGCTGAGTCACTGGCAGGGCAGCGCGCGTGTGGGAAGGGGCGGAGGGAGTGCGGCCGGCGGGCGGGCGGGGCGCTGGGCTCAGCCCGGCC
GCAGGTGACCCGGAGGCCCTCGCCGCCCGCGGCGCCCCGAGCGCTTTGTGAGCAGATGCGGAGCCGAGTGGAGGGCGCGAGCCAGATGCGGGGCGAC
AGCTGACTTGCTGAGAGGAGGCGGGGAGGCGCGGAGCGCGCGTGTGGTCCTTGCGCCGCTGACTTCTCCACTGGTTCCTGGGCACCGAAAG>ENSG000
00105976|1|MET
GCTGTTAAATTGAAAATTGACTTAGCCAACCGAGAGACAAGCATCTTCAGTTACCGTGAAGATCCCATTGTCTATGAAATTCATCCAACCAAATCTTTTATT
AG>ENSG00000105976|1|MET
AGGTGACCCGGAGGCCCTCGCCGCCCGCGGCGCCCCGAGCGCTTTGTGAGCAGATGCGGAGCCGAGTGGAGGGCGCGAGCCAGATGCGGGGCGACAG
CTGACTTGCTGAGAGGAGGCGGGGAGGCGCGGAGCGCGCGTGTGGTCCTTGCGCCGCTGACTTCTCCACTGGTTCCTGGGCACCGAAAG>ENSG000001
05976|2|MET
GCAGAAAATGTGCTAGATTGGAGGTGAAGACCCTGGAGCCAGAGAGCCTAGGCTTAGTCCTAGCCCTGCACTGAAG>ENSG0000105976|2|MET

GGTGGTGATGAAGAGTAAATCAAAGAGTTTAGCAGAATGCTTCCCATATG>ENSG00000105976|2|MET
ATAAACCTCTCATAATGAAGGCCCCCGCTGTGCTTGCACCTGGCATCCTCGTGCTCCTGTTTACCTTGGTGCAGAGGAGCAATGGGGAGTGTAAAGAGGC
ACTAGCAAAGTCCGAGATGAATGTGAATATGAAGTATCAGCTTCCCAACTTCACCGCGGAAACACCCATCCAGAATGTCATTCTACATGAGCATCACATTT
TCCTTGGTGCCACTAACTACATTTATGTTTTAAATGAGGAAGACCTTCAGAAGGTTGCTGAGTACAAGACTGGGCCTGTGCTGGAACACCCAGATTGTTTC
CCATGTCAGGACTGCAGCAGCAAAGCCAATTTATCAGGAGGTGTTTGGAAAGATAACATCAACATGGCTCTAGTTGTCGACACCTACTATGATGATCAACT
CATTAGCTGTGGCAGCGTCAACAGAGGGACCTGCCAGCGACATGTCTTTCCCCACAATCATACTGCTGACATACAGTCGGAGGTTCACTGCATATTCTCCC
CACAGATAGAAGAGCCCAGCCAGTGTCCTGACTGTGTGGTGAGCGCCCTGGGAGCCAAAGTCCTTTCATCTGTAAAGGACCGGTTCATCAACTTCTTTGTA
GGCAATACCATAAATTCTTCTTATTTCCCAGATCATCCATTGCATTCGATATCAGTGAGAAGGCTAAAGGAAACGAAAGATGGTTTTATGTTTTTGACGGAC
CAGTCCTACATTGATGTTTTACCTGAGTTCAGAGATTCTTACCCCATTAAGTATGTCCATGCCTTTGAAAGCAACAATTTTATTTACTTCTTGACGGTCCAAA
GGGAAACTCTAGATGCTCAGACTTTTCACACAAGAATAATCAGGTTCTGTTCCATAAACTCTGGATTGCATTCCTACATGGAAATGCCTCTGGAGTGTATTC
TCACAGAAAAGAGAAAAAAGAGATCCACAAAGAAGGAAGTGTTTAATATACTTCAGGCTGCGTATGTCAGCAAGCCTGGGGCCCAGCTTGCTAGACAAA
TAGGAGCCAGCCTGAATGATGACATTCTTTTCGGGGTGTTCGCACAAAGCAAGCCAGATTCTGCCGAACCAATGGATCGATCTGCCATGTGTGCATTCCCT
ATCAAATATGTCAACGACTTCTTCAACAAGATCGTCAACAAAAACAATGTGAGATGTCTCCAGCATTTTTACGGACCCAATCATGAGCACTGCTTTAATAGG
>ENSG00000105976|3;2|MET
ACACTTCTGAGAAATTCATCAGGCTGTGAAGCGCGCCGTGATGAATATCGAACAGAGTTTACCACAGCTTTGCAGCGCGTTGACTTATTCATGGGTCAATT
CAGCGAAGTCCTCTTAACATCTATATCCACCTTCATTAAAGGAGACCTCACCATAGCTAATCTTGGGACATCAGAGGGTCGCTTCATGCAG>ENSG000001
05976|3|MET
ATAAACCTCTCATAATGAAGGCCCCCGCTGTGCTTGCACCTGGCATCCTCGTGCTCCTGTTTACCTTGGTGCAGAGGAGCAATGGGGAGTGTAAAGAGGC
ACTAGCAAAGTCCGAGATGAATGTGAATATGAAGTATCAGCTTCCCAACTTCACCGCGGAAACACCCATCCAGAATGTCATTCTACATGAGCATCACATTT
TCCTTGGTGCCACTAACTACATTTATGTTTTAAATGAGGAAGACCTTCAGAAGGTTGCTGAGTACAAGACTGGGCCTGTGCTGGAACACCCAGATTGTTTC
CCATGTCAGGACTGCAGCAGCAAAGCCAATTTATCAGGAGGTGTTTGGAAAGATAACATCAACATGGCTCTAGTTGTCGACACCTACTATGATGATCAACT
CATTAGCTGTGGCAGCGTCAACAGAGGGACCTGCCAGCGACATGTCTTTCCCCACAATCATACTGCTGACATACAGTCGGAGGTTCACTGCATATTCTCCC
CACAGATAGAAGAGCCCAGCCAGTG>ENSG00000105976|3|MET
ACACTTCTGAGAAATTCATCAGGCTGTGAAGCGCGCCGTGATGAATATCGAACAGAGTTTACCACAGCTTTGCAGCGCGTTGACTTATTCATGGGTCAATT
CAGCGAAGTCCTCTTAACATCTATATCCACCTTCATTAAAGGAGACCTCACCATAGCTAATCTTGGGACATCAGAGGGTCGCTTCATGCAG>ENSG000001
05976|3|MET
ATCAGTTTCCTAATTCATCTCAGAACGGTTCATGCCGACAAGTGCAGTATCCTCTGACAGACATGTCCCCCATCCTAACTAGTGGGGACTCTGATATATCCA
GTCCATTACTGCAAAATACTGTCCACATTGACCTCAGTGCTCTAAATCCAGAGCTGGTCCAGGCAGTGCAGCATGTAGTGATTGGGCCCA>ENSG0000010
5976|3;4|MET
GTTGTGGTTTCTCGATCAGGACCATCAACCCCTCATGTGAATTTTCTCCTGGACTCCCATCCAGTGTCTCCAGAAGTGATTGTGGAGCATACATTAAACCAA
AATGGCTACACACTGGTTATCACTGGGAAGAAG>ENSG00000105976|4|MET
GGAAATGATATTGACCCTGAAGCAGTTAAAGGTGAAGTGTTAAAAGTTGGAAATAAGAGCTGTGAGAATATACACTTACATTCTGAAGCCGTTTTATGCA
CGGTCCCCAATGACCTGCTGAAATTGAACAGCGAGCTAAATATAGAGGTGGGATTCCTGCATTCCTCTCATGATGTAAATAAGGAAGCCAGTGTAATTATG
TTATTCTCAGGCTTAAAATAAATCATTAAAGCTCATTTATGTGTGGGTTTTGGCTCATCAACTC>ENSG00000105976|4|MET
GTTGTGGTTTCTCGATCAGGACCATCAACCCCTCATGTGAATTTTCTCCTGGACTCCCATCCAGTGTCTCCAGAAGTGATTGTGGAGCATACATTAAACCAA
AATGGCTACACACTGGTTATCACTGGGAAGAAG>ENSG00000105976|5;4|MET
ATCACGAAGATCCCATTGAATGGCTTGGGCTGCAGACATTTCCAGTCCTGCAGTCAATGCCTCTCTGCCCCACCCTTTGTTCAGTGTGGCTGGTGCCACGAC
AAATGTGTGCGATCGGAGGAATGCCTGAGCGGGACATGGACTCAACAGATCTGTCTGCCTGCAATCTACAAG>ENSG00000105976|5|MET
ATCACGAAGATCCCATTGAATGGCTTGGGCTGCAGACATTTCCAGTCCTGCAGTCAATGCCTCTCTGCCCCACCCTTTGTTCAGTGTGGCTGGTGCCACGAC
AAATGTGTGCGATCGGAGGAATGCCTGAGCGGGACATGGACTCAACAGATCTGTCTGCCTGCAATCTACAAG>ENSG00000105976|5;6|MET
GTTTTCCCAAATAGTGCACCCCTTGAAGGAGGGACAAGGCTGACCATATGTGGCTGGGACTTTGGATTTCGGAGGAATAATAAATTTGATTTAAAGAAAA
CTAGAGTTCTCCTTGGAAATGAGAGCTGCACCTTGACTTTAAGTGAGAGCACGATGAATAC>ENSG00000105976|6|MET
GTTTTCCCAAATAGTGCACCCCTTGAAGGAGGGACAAGGCTGACCATATGTGGCTGGGAC>ENSG00000105976|7;6|MET
ATTGAAATGCACAGTTGGTCCTGCCATGAATAAGCATTTCAATATGTCCATAATTATTTCAAATGGCCACGGGACAACACAATACAGTACATTCTCCTATGT
G>ENSG00000105976|8;7|MET
GATCCTGTAATAACAAGTATTTCGCCGAAATACGGTCCTATGGCTGGTGGCACTTTACTTACTTTAACTGGAAATTACCTAAACAGTGGGAATTCTAGACAC
ATTTCAATTGGTGGAAAAACATGTACTTTAAAAAG>ENSG00000105976|8;9|MET
TGTGTCAAACAGTATTCTTGAATGTTATACCCCAGCCCAAACCATTTCAACTGAGTTTGCTGTTAAATTGAAAATTGACTTAGCCAACCGAGAGACAAGCAT
CTTCAGTTACCGTGAAGATCCCATTGTCTATGAAATTCATCCAACCAAATCTTTTATTAG>ENSG00000105976|9|MET
GCATGTCAACATCGCTCTAATTCAGAGATAA>ENSG00000105976|10|MET
TACTTGGTGGAAAGAACCTCTCAACATTGTCAGTTTTCTATTTTGCTTTGCCAGTGGTGGGAGCACAATAACAGGTGTTGGGAAAAACCTGAATTCAGTTA
GTGTCCCGAGAATGGTCATAAATGTGCATGAAGCAGGAAGGAACTTTACAGTG>ENSG00000105976|10;2|MET
TGGTGGGAGCACAATAACAGGTGTTGGGAAAAACCTGAATTCAGTTAGTGTCCCGAGAATGGTCATAAATGTGCATGAAGCAGGAAGGAACTTTACAGT
G>ENSG00000105976|11;3|MET
GCATGTCAACATCGCTCTAATTCAGAGATAATCTGTTGTACCACTCCTTCCCTGCAACAGCTGAATCTGCAACTCCCCCTGAAAACCAAAGCCTTTTTCATGT
TAGATGGGATCCTTTCCAAATACTTTGATCTCATTTATGTACATAATCCTGTGTTTAAGCCTTTTGAAAAGCCAGTGATGATCTCAATGGGCAATGAAAATG
TACTGGAAATTAAG>ENSG00000105976|12|MET
GGAAATGATATTGACCCTGAAGCAGTTAAAGGTGAAGTGTTAAAAGTTGGAAATAAGAGCTGTGAGAATATACACTTACATTCTGAAGCCGTTTTATGCA
CGGTCCCCAATGACCTGCTGAAATTGAACAGCGAGCTAAATATAGAG>ENSG00000105976|13;2|MET
TGGAAGCAAGCAATTTCTTCAACCGTCCTTGGAAAAGTAATAGTTCAACCAGATCAGAATTTCACAGGATTGATTGCTGGTGTTGTCTCAATATCAACAGC
ACTGTTATTACTACTTGGGTTTTTCCTGTGGCTGAAAAAGAGAAAGCAAATTAAAG>ENSG00000105976|14|MET
ATCTGGGCAGTGAATTAGTTCGCTACGATGCAAGAGTACACACTCCTCATTTGGATAGGCTTGTAAGTGCCCGAAGTGTAAGCCCAACTACAGAAATGGT
TTCAAATGAATCTGTAGACTACCGAGCTACTTTTCCAGAAG>ENSG00000105976|15|MET
ATCAGTTTCCTAATTCATCTCAGAACGGTTCATGCCGACAAGTGCAGTATCCTCTGACAGACATGTCCCCCATCCTAACTAGTGGGGACTCTGATATATCCA
GTCCATTACTGCAAAATACTGTCCACATTGACCTCAGTGCTCTAAATCCAGAGCTGGTCCAGGCAGTGCAGCATGTAGTGATTGGGCCCAGTAGCCTGATT
GTGCATTTCAATGAAGTCATAGGAAGAG>ENSG00000105976|16|MET
GGCATTTTGGTTGTGTATATCATGGGACTTTGTTGGACAATGATGGCAAGAAAATTCACTGTGCTGTGAAATCCTTGAACA>ENSG00000105976|17|M
ET
GAATCACTGACATAGGAGAAGTTTCCCAATTTCTGACCGAGGGAATCATCATGAAAGATTTTAGTCATCCCAATGTCCTCTCGCTCCTGGGAATCTGCCTGC
GAAGTGAAGGGTCTCCGCTGGTGGTCCTACCATACATGAAACATGGAGATCTTCGAAATTTCATTCGAAATGAGACTCAT>ENSG00000105976|18|ME
T

AATCCAACTGTAAAAGATCTTATTGGCTTTGGTCTTCAAGTAGCCAAAGGCATGAAATATCTTGCAAGCAAAAAGTTTGTCCACAGAGACTTGGCTGCAAG
AAACTGTAT>ENSG00000105976|19|MET
GCTGGATGAAAAATTCACAGTCAAGGTTGCTGATTTTGGTCTTGCCAGAGACATGTATGATAAAGAATACTATAGTGTACACAACAAAACAGGTGCAAAG
CTGCCAGTGAAGTGGATGGCTTTGGAAAGTCTGCAAACTCAAAAGTTTACCACCAAGTCAGATGTG>ENSG00000105976|20|MET
TGGTCCTTTGGCGTGCTCCTCTGGGAGCTGATGACAAGAGGAGCCCCACCTTATCCTGACGTAAACACCTTTGATATAACTGTTTACTTGTTGCAAGGGAG
AAGACTCCTACAACCCGAATACTGCCCAGACCCCTT>ENSG00000105976|21|MET
ATATGAAGTAATGCTAAAATGCTGGCACCCTAAAGCCGAAATGCGCCCATCCTTTTCTGAACTGGTGTCCCGGATATCAGCGATCTTCTCTACTTTCATTGG
GGAGCACTATGTCCATGTGAACGCTACTTATGTGAACGTAAAATGTGTCGCTCCGTATCCTTCTCTGTTGTCATCAGAAGATAACGCTGATGATGAGGTGG
ACACACGACCAGCCTCCTTCTGGGAGACATCATAGTGCTAGTACTATGTCAAAGCAACAGTCCACACTTTGTCCAATGGTTTTTTCACTGCCTGACCTTTAA
AAGGCCATCGATATTCTTTGCTCTTGCCAAAATTGCACTATTATAGGACTTGTATTGTTATTTAAATTACTGGATTCTAAGGAATTTCTTATCTGACAGAGCA
TCAGAACCAGAGGCTTGGTCCCACAGGCCACGGACCAATGGCCTGCAGCCGTGACAACACTCCTGTCATATTGGAGTCCAAAACTTGAATTCTGGGTTGA
ATTTTTTAAAAATCAGGTACCACTTGATTTCATATGGGAAATTGAAGCAGGAAATATTGAGGGCTTCTTGATCACAGAAAACTCAGAAGAGATAGTAATGC
TCAGGACAGGAGCGGCAGCCCCAGAACAGGCCACTCATTTAGAATTCTAGTGTTTCAAAACACTTTTGTGTGTTGTATGGTCAATAACATTTTTCATTACTG
ATGGTGTCATTCACCCATTAGGTAAACATTCCCTTTTAAATGTTTGTTTGTTTTTTGAGACAGGATCTCACTCTGTTGCCAGGGCTGTAGTGCAGTGGTGTG
ATCATAGCTCACTGCAACCTCCACCTCCCAGGCTCAAGCCTCCCGAATAGCTGGGACTACAGGCGCACACCACCATCCCCGGCTAATTTTTGTATTTTTTGT
AGAGACGGGGTTTTGCCATGTTGCCAAGGCTGGTTTCAAACTCCTGGACTCAAGAAATCCACCCACCTCAGCCTCCCAAAGTGCTAGGATTACAGGCATG
AGCCACTGCGCCCAGCCCTTATAAATTTTTGTATAGACATTCCTTTGGTTGGAAGAATATTTATAGGCAATACAGTCAAAGTTTCAAAATAGCATCACACAA
AACATGTTTATAAATGAACAGGATGTAATGTACATAGATGACATTAAGAAAATTTGTATGAAATAATTTAGTCATCATGAAATATTTAGTTGTCATATAAAA
ACCCACTGTTTGAGAATGATGCTACTCTGATCTAATGAATGTGAACATGTAGATGTTTTGTGTGTATTTTTTTAAATGAAAACTCAAAATAAGACAAGTAAT
TTGTTGATAAATATTTTTAAAGATAACTCAGCATGTTTGTAAAGCAGGATACATTTTACTAAAAGGTTCATTGGTTCCAATCACAGCTCATAGGTAGAGCAA
AGAAAGGGTGGATGGATTGAAAAGATTAGCCTCTGTCTCGGTGGCAGGTTCCCACCTCGCAAGCAATTGGAAACAAAACTTTTGGGGAGTTTTATTTTGC
ATTAGGGTGTGTTTTATGTTAAGCAAAACATACTTTAGAAACAAATGAAAAAGGCAATTGAAAATCCCAGCTATTTCACCTAGATGGAATAGCCACCCTGA
GCAGAACTTTGTGATGCTTCATTCTGTGGAATTTTGTGCTTGCTACTGTATAGTGCATGTGGTGTAGGTTACTCTAACTGGTTTTGTCGACGTAAACATTTA
AAGTGTTATATTTTTTATAAAAATGTTTATTTTTAATGATATGAGAAAAAATTTTGTTAGGCCACAAAAACACTGCACTGTGAACATTTTAGAAAAGGTATGT
CAGACTGGGATTAATGACAGCATGATTTTCAATGACTGTAAATTGCGATAAGGAAATGTACTGATTGCCAATACACCCCACCCTCATTACATCATCAGGAC
TTGAAGCCAAGGGTTAACCCAGCAAGCTACAAAGAGGGTGTGTCACACTGAAACTCAATAGTTGAGTTTGGCTGTTGTTGCAGGAAAATGATTATAACTA
AAAGCTCTCTGATAGTGCAGAGACTTACCAGAAGACACAAGGAATTGTACTGAAGAGCTATTACAATCCAAATATTGCCGTTTCATAAATGTAATAAGTAA
TACTAATTCACAGAGTATTGTAAATGGTGGATGACAAAAGAAAATCTGCTCTGTGGAAAGAAAGAACTGTCTCTACCAGGGTCAAGAGCATGAACGCATC
AATAGAAAGAACTCGGGGAAACATCCCATCAACAGGACTACACACTTGTATATACATTCTTGAGAACACTGCAATGTGAAAATCACGTTTGCTATTTATAA
ACTTGTCCTTAGATTAATGTGTCTGGACAGATTGTGGGAGTAAGTGATTCTTCTAAGAATTAGATACTTGTCACTGCCTATACCTGCAGCTGAACTGAATGG
TACTTCGTATGTTAATAGTTGTTCTGATAAATCATGCAATTAAAGTAAAGTGATGCAACATCTTGTA>ENSG00000105976|21|MET
ATATGAAGTAATGCTAAAATGCTGGCACCCTAAAGCCGAAATGCGCCCATCCTTTTCTGAACTGGTGTCCCGGATATCAGCGATCTTCTCTACTTTCATTGG
GGAGCACTATGTCCATGTGAACGCTACTTATGTGAACGTAAAATGTGTCGCTCCGTATCCTTCTCTGTTGTCATCAGAAGATAACGCTGATGATGAGGTGG
ACACACGACCAGCCTCCTTCTGGGAGACATCATAGTGCTAGTACTATGTCAAAGCAACAGTCCACACTTTGTCCAATGGTTTTTTCACTGCCTGACCTTTAA
AAGGCCATCGATATTCTTTGCTCTTGCCAAAATTGCACTATTATAGGACTTGTATTGTTATTTAAATTACTGGATTCTAAGGAATTTCTTATCTGACAGAGCA
TCAGAACCAGAGGCTTGGTCCCACAGGCCACGGACCAATGGCCTGCAGCCGTGACAACACTCCTGTCATATTGGAGTCCAAAACTTGAATTCTGGGTTGA
ATTTTTTAAAAATCAGGTACCACTTGATTTCATATGGGAAATTGAAGCAGGAAATATTGAGGGCTTCTTGATCACAGAAAACTCAGAAGAGATAGTAATGC
TCAGGACAGGAGCGGCAGCCCCAGAACAGGCCACTCATTTAGAATTCTAGTGTTTCAAAACACTTTTGTGTGTTGTATGGTCAATAACATTTTTCATTACTG
ATGGTGTCATTCACCCATTAGGTAAACATTCCCTTTTAAATGTTTGTTTGTTTTTTGAGACAGGATCTCACTCTGTTGCCAGGGCTGTAGTGCAGTGGTGTG
ATCATAGCTCACTGCAACCTCCACCTCCCAGGCTCAAGCCTCCCGAATAGCTGGGACTACAGGCGCACACCACCATCCCCGGCTAATTTTTGTATTTTTTGT
AGAGACGGGGTTTTGCCATGTTGCCAAGGCTGGTTTCAAACTCCTGGACTCAAGAAATCCACCCACCTCAGCCTCCCAAAGTGCTAGGATTACAGGCATG
AGCCACTGCGCCCAGCCCTTATAAATTTTTGTATAGACATTCCTTTGGTTGGAAGAATATTTATAGGCAATACAGTCAAAGTTTCAAAATAGCATCACACAA
AACATGTTTATAAATGAACAGGATGTAATGTACATAGATGACATTAAGAAAATTTGTATGAAATAATTTAGTCATCATGAAATATTTAGTTGTCATATAAAA
ACCCACTGTTTGAGAATGATGCTACTCTGATCTAATGAATGTGAACATGTAGATGTTTTGTGTGTATTTTTTTAAATGAAAACTCAAAATAAGACAAGTAAT
TTGTTGATAAATATTTTTAAAGATAACTCAGCATGTTTGTAAAGCAGGATACATTTTACTAAAAGGTTCATTGGTTCCAATCACAGCTCATAGGTAGAGCAA
AGAAAGGGTGGATGGATTGAAAAGATTAGCCTCTGTCTCGGTGGCAGGTTCCCACCTCGCAAGCAATTGGAAACAAAACTTTTGGGGAGTTTTATTTTGC
ATTAGGGTGTGTTTTATGTTAAGCAAAACATACTTTAGAAACAAATGAAAAAGGCAATTGAAAATCCCAGCTATTTCACCTAGATGGAATAGCCACCCTGA
GCAGAACTTTGTGATGCTTCATTCTGTGGAATTTTGTGCTTGCTACTGTATAGTGCATGTGGTGTAGGTTACTCTAACTGGTTTTGTCGACGTAAACATTTA
AAGTGTTATATTTTTTATAAAAATGTTTATTTTTAATGATATGAGAAAAAATTTTGTTAGGCCACAAAAACACTGCACTGTGAACATTTTAGAAAAGGTATGT
CAGACTGGGATTAATGACAGCATGATTTTCAATGACTGTAAATTGCGATAAGGAAATGTACTGATTGCCAATACACCCCACCCTCATTACATCATCAGGAC
TTGAAGCCAAGGGTTAACCCAGCAAGCTACAAAGAGGGTGTGTCACACTGAAACTCAATAGTTGAGTTTGGCTGTTGTTGCAGGAAAATGATTATAACTA
AAAGCTCTCTGATAGTGCAGAGACTTACCAGAAGACACAAGGAATTGTACTGAAGAGCTATTACAATCCAAATATTGCCGTTTCATAAATGTAATAAGTAA
TACTAATTCACAGAGTATTGTAAATGGTGGATGACAAAAGAAAATCTGCTCTGTGGAAAGAAAGAACTGTCTCTACCAGGGTCAAGAGCATGAACGCATC
AATAGAAAGAACTCGGGGAAACATCCCATCAACAGGACTACACACTTGTATATACATTCTTGAGAACACTGCAATGTGAAAATCACGTTTGCTATTTATAA
ACTTGTCCTTAGATTAATGTGTCTGGACAGATTGTGGGAGTAAGTGATTCTTCTAAGAATTAGATACTTGTCACTGCCTATACCTGCAGCTGAACTGAATGG
TACTTCGTATGTTAATAGTTGTTCTGATAAATCATGCAATTAAAGTAAAGTGATGCAA

Supplementary Table 4S: Samples characterized by unexpected coverage peak in intron 2.

Tissue	Sample	# incorrectly paired MET reads	# MET reads paired with LINE1 sequence
Cervix (uterus)	8f050c2c-c83b-4d26-8038-f3ca13e09ced	121	1
Cervix (uterus)	14d39df5-73b7-40aa-9d77-efbaad8a23c5	8	0
Cervix (uterus)	4385ae24-fd72-491a-bfd0-484feacd5650	452	3
Cervix (uterus)	449380d4-f521-4d94-8923-811375f79bd7	163	1
Cervix (uterus)	5501872f-ee40-41f6-b22c-ada59e993b3e	125	3
Cervix (uterus)	c751ab77-8d6d-4b2b-9345-a3ba05bb259e	158	0
Corpus (uterus)	39bb1145-0d48-4968-92ec-6c91484edc87	192	6
Corpus (uterus)	707cd6a3-b7de-47af-a317-5dca82014b1a	77	4
Corpus (uterus)	815fc29e-72f6-493b-9ab6-c91c5f12a1a6	306	4
Corpus (uterus)	c21a8f3c-584a-44d7-8902-3c73d4b09b99	0	0
Heart/Mediastinum/Pleura	7f003fdd-09aa-4e42-a515-e2967c20b1e3	28	1
Kidney	164e9f2a-c06f-444c-a27c-07585e0da7a7	120	2
Kidney	947aaaf2-8073-4816-9877-04dcce0a1957	657	4
Kidney	70502d21-3796-4041-82ef-7bb04dd28d11	31	0
Skin	382dea5d-70fe-4a4f-b223-88264c9b3804	17	0

Supplementary Table 5S: Samples characterized by the presence of a MET read(s) paired with a read mapping in LINE1 sequence.

Tissue	Sample	position in MET locus	Location in MET locus
Kidney	164e9f2a-c06f-444c-a27c-07585e0da7a7	116793365	intron 19
Kidney	164e9f2a-c06f-444c-a27c-07585e0da7a7	116793365	intron 19
Corpus (uterus)	39bb1145-0d48-4968-92ec-6c91484edc87	116695965	intron 1
Corpus (uterus)	39bb1145-0d48-4968-92ec-6c91484edc87	116695967	intron 1
Corpus (uterus)	39bb1145-0d48-4968-92ec-6c91484edc87	116695967	intron 1
Corpus (uterus)	39bb1145-0d48-4968-92ec-6c91484edc87	116713208	intron 2
Corpus (uterus)	39bb1145-0d48-4968-92ec-6c91484edc87	116759968	intron 10
Corpus (uterus)	39bb1145-0d48-4968-92ec-6c91484edc87	116759968	intron 10
Cervix (uterus)	4385ae24-fd72-491a-bfd0-484feacd5650	116694835	intron 1
Cervix (uterus)	4385ae24-fd72-491a-bfd0-484feacd5650	116751947	intron 5
Cervix (uterus)	4385ae24-fd72-491a-bfd0-484feacd5650	116793744	intron 19
Cervix (uterus)	449380d4-f521-4d94-8923-811375f79bd7	116713208	intron 2
Cervix (uterus)	5501872f-ee40-41f6-b22c-ada59e993b3e	116685410	intron 1
Cervix (uterus)	5501872f-ee40-41f6-b22c-ada59e993b3e	116695967	intron 1
Cervix (uterus)	5501872f-ee40-41f6-b22c-ada59e993b3e	116765089	intron 11
Corpus (uterus)	707cd6a3-b7de-47af-a317-5dca82014b1a	116716185	intron 2
Corpus (uterus)	707cd6a3-b7de-47af-a317-5dca82014b1a	116716189	intron 2
Corpus (uterus)	707cd6a3-b7de-47af-a317-5dca82014b1a	116716189	intron 2
Corpus (uterus)	707cd6a3-b7de-47af-a317-5dca82014b1a	116716189	intron 2
Heart/Mediastinum/Pleura	7f003fdd-09aa-4e42-a515-e2967c20b1e3	116713208	intron 2
Corpus (uterus)	815fc29e-72f6-493b-9ab6-c91c5f12a1a6	116694778	intron 1
Corpus (uterus)	815fc29e-72f6-493b-9ab6-c91c5f12a1a6	116694778	intron 1
Corpus (uterus)	815fc29e-72f6-493b-9ab6-c91c5f12a1a6	116751826	intron 5
Corpus (uterus)	815fc29e-72f6-493b-9ab6-c91c5f12a1a6	116751827	intron 5
Cervix (uterus)	8f050c2c-c83b-4d26-8038-f3ca13e09ced	116751954	intron 5
Kidney	947aaaf2-8073-4816-9877-04dcce0a1957	116793487	intron 19
Kidney	947aaaf2-8073-4816-9877-04dcce0a1957	116796727	exon 21
Kidney	947aaaf2-8073-4816-9877-04dcce0a1957	116796727	exon 21
Kidney	947aaaf2-8073-4816-9877-04dcce0a1957	116796808	exon 21

Supplementary Table 6S: Samples in TCGA bronchus and lung predicted as METΔ14 using different training configurations. METΔ14 score ≤ 0.1 predicts a skipped event.

CNN training	sample	METΔ14 score	NN quality score	MET reads	sample mapped reads	sample unmapped reads
16 kmer counts	0f8f62da-daf0-4a59-9740-b03bd0e0ea3b	0.004186183	5951	69446926.5	1678773	FALSE
16 kmer counts	4f7de6bd-55ea-43c3-a04a-1c57d647d4d3	0.006279737	34087	44562703.5	855013.5	TRUE
16 kmer counts	7dde9229-a172-466f-b4f5-12508fb3938a	0.054982454	17090	113470656	2020366	FALSE
16 kmer counts	9b056fb3-407d-4adc-9720-25b9d3055be7	0.005349129	11391	78987772	1998091.5	FALSE
16 kmer counts	b7818aa6-af2b-4eda-967f-3db7b30749fb	0.026182353	12301	96460534.5	4762668	FALSE

16 kmer counts	c18f5c1b-4597-4e56-ad21-bafd975fd94e	0.05905375	12301	96460534.5	4762668	FALSE
16 kmer counts	ce9c0d52-542b-4586-99b0-4c409582e76d	0.011204243	39918	124595862.5	2162904.5	FALSE
16 kmer counts	d34a6605-5d94-4fea-a455-7e9e70495953	0.013193339	39918	124595862.5	2162904.5	FALSE
16 kmer counts	e6d1567b-49d5-428b-a3d7-cc82481ae1d8	0.053991586	5302	74718854.5	2951197.5	FALSE
16 kmer counts	ffef03e7-ce56-408e-a6d6-2357f597da28	0.09233853	24691	51135944.5	830894	FALSE
13-15 MET k-mer counts frequency	92650f66-f8b3-49c8-a1fc-04fba0ffb6fe	1.32024E-05	32307	60202242	1021761	TRUE
13-15 MET k-mer counts frequency	4f7de6bd-55ea-43c3-a04a-1c57d647d4d3	1.46925E-05	34087	44562703.5	855013.5	TRUE
13-15 MET k-mer counts frequency	15750b18-0522-4186-8809-e7e3e7e4f6f7	1.67191E-05	28130	87516179.5	3472464.5	TRUE
13-15 MET k-mer counts frequency	eaf344c1-a5e8-4893-b17e-4a0dbb1bf2de	1.75536E-05	19083	41267020.5	752423	TRUE
13-15 MET k-mer counts frequency	56b38e12-3495-4419-8df6-d3504b190357	1.92225E-05	44070	136788143	43362420	TRUE
13-15 MET k-mer counts frequency	654c93db-8b88-4619-ae04-e4c561133bc3	2.13683E-05	114696	60171126	990399.5	TRUE
13-15 MET k-mer counts frequency	509b1e8b-8e1e-4d27-b9c7-a13942ae5dc9	2.97427E-05	127554	68401183	1726606	TRUE
13-15 MET k-mer counts frequency	be2ba5b6-5652-4daa-a29f-e4db98262008	6.41346E-05	58379	65644230.5	1644546	TRUE
13-15 MET k-mer counts frequency	911fdc9d-3de5-493d-98c7-f567072ca1d5	7.41184E-05	34418	78565359	2271496	TRUE
13-15 MET k-mer counts frequency	8cac0361-6973-4ae6-a34a-58f355085ec0	0.000107229	19455	59264871	2258010	TRUE
13-15 MET k-mer counts frequency	9d5fde39-1fc9-4079-b095-bdc94bbb3679	0.000151336	26170	60892059.5	1328162.5	TRUE
13-15 MET k-mer counts frequency	5f7c9fc6-f894-4dce-98b0-a9d3a36392e8	0.001333982	28641	106888363.5	3169442.5	TRUE
13-15 MET k-mer counts frequency	010a6f81-5601-4fd6-ad42-4d5670d48ff1	0.007802546	139031	86536796	1746345	TRUE
13-15 MET k-mer counts frequency	2a7a996d-89ab-4717-831f-788461559037	0.014151275	7560	43568960.5	563888	TRUE
13-15 MET k-mer counts frequency	c9032291-b671-4280-96d2-7a5806e66886	0.04142213	59071	90868832	1714512	TRUE
13-15 MET k-mer counts frequency	e210c2a9-40c7-4f6e-9e5a-d6840c2e3a5c	0.0564214	8684	35173297.5	507258.5	TRUE
13-15 MET coverage frequency	4f7de6bd-55ea-43c3-a04a-1c57d647d4d3	0.064038634	34087	44562703.5	855013.5	TRUE
13-15 MET coverage frequency	56b38e12-3495-4419-8df6-d3504b190357	0.07843056	44070	136788143	43362420	TRUE
13-15 MET coverage frequency	15750b18-0522-4186-8809-e7e3e7e4f6f7	0.078582495	28130	87516179.5	3472464.5	TRUE
13-15 MET coverage frequency	92650f66-f8b3-49c8-a1fc-04fba0ffb6fe	0.08228013	32307	60202242	1021761	TRUE
13-15 MET coverage frequency	654c93db-8b88-4619-ae04-e4c561133bc3	0.0826129	114696	60171126	990399.5	TRUE
13-15 MET coverage frequency	eaf344c1-a5e8-4893-b17e-4a0dbb1bf2de	0.08437839	19083	41267020.5	752423	TRUE
13-15 MET coverage frequency	509b1e8b-8e1e-4d27-b9c7-a13942ae5dc9	0.09332588	127554	68401183	1726606	TRUE
13-15 MET coverage frequency	be2ba5b6-5652-4daa-a29f-e4db98262008	0.09363499	58379	65644230.5	1644546	TRUE
